# Editorial: Role of fibroblast in cancer

**DOI:** 10.3389/fmolb.2025.1574538

**Published:** 2025-02-27

**Authors:** Xuezhen Zeng, Man Liu

**Affiliations:** ^1^ Institute of Precision Medicine, the First Affiliated Hospital, Sun Yat-sen University, Guangzhou, Guangdong, China; ^2^ Department of Gastroenterology, The First Affiliated Hospital, Sun Yat-sen University, Guangzhou, Guangdong, China

**Keywords:** fibroblast, tumor microenvironment (TME, ), cancer, cancer associated fibroblast (CAF), therapy

Cancer-associated fibroblasts (CAFs) are a heterogeneous population of cells with diverse origins, phenotypes, and functions, and the major component of the tumor microenvironment (TME) which play critical roles in tumor initiation, progression, and metastasis (Curtis et al., 2019; Lavie et al., 2022). CAFs are activated fibroblasts that exhibit an altered phenotype and secrete various growth factors, cytokines, chemokines and extracellular matrix (ECM) proteins to promote cancer cell proliferation, survival, angiogenesis and shape the tumor immune microenvironment (Arpinati et al., 2024). Increasing evidence showed that CAFs can suppress anti-tumor immune responses by recruiting immunosuppressive cells to inhibit the activation and function of cytotoxic T cells and natural killer (NK) cells (Kumar et al., 2017; Francescone et al., 2021). CAFs are therefore a key determinant in the malignant progression of cancer. Targeting CAFs has emerged as a promising strategy for cancer therapy (Broz et al., 2024). This Research Topic brings together cutting-edge research and review on the pivotal role of fibroblast in cancer and therapeutic potential.

In this section, Cheng et al. analyzed 5,190 publications related to CAFs by bibliometric tools such as VOSviewer and CiteSpace. They found that China and the United States were the leading contributors in terms of publications, funding agencies, and international collaborations. Among all the CAF-associated studies, breast, colorectal, pancreatic, prostate and gastric cancer were the top 5 studied cancers. And CAFs have been demonstrated to correlate with immune cell infiltration (including T cells, B cells, tumor-associated macrophages), antitumor immunity or immunosuppression, the efficacy of immunotherapy (PD-L1), cancer cell stemness, and DNA methylation, etc. Several genes such as TGFB1, IL-6, TNF, TP53, and VEGFA, and pathways such as HIF-1 and Toll-like receptor signaling have been highlighted in these studies. This article provides a overview of the CAF-related studies, highlighting current trends and future directions with a focus on advancements in the tumor immune microenvironment.

Immunotherapy has revolutionized the treatment of cancers, with its effectiveness significantly influenced by the tumor microenvironment. Cancer-associated fibroblasts (CAFs) have emerged as promising targets to enhance the efficacy of immunotherapy, but specific CAF marker is lacking. Xia et al. has developed a CAF Angiogenesis Prognostic Score (CAPS) system in gastric cancer (GC) patients. Several differentially expressed genes associated with CAF-angiogenesis were identified, including THBS1, SPARC, EDNRA, and VCAN, which were used to establish the CAPS. RT-qPCR experiment, GEO datasets, and the HPA database were utilized to validate the CAPS gene expression. In addition, high-CAPS correlated with shorter overall survival and a tendency toward immune escape and reduced immunotherapy efficacy. Thus, CAPS serves as an independent predictor of GC prognosis and immunotherapy efficacy.

In prostate cancer (PCa), Torres et al. unraveled that PCa associated fibroblasts (PCAFs) displayed different metabolic and functional profile compared to normal prostate fibroblasts (PFs). PCAFs exhibited reduced ECM degradation activity under normoxic condition compared to PFs. In addition, PCAFs and PFs displayed distinct metabolic characteristics under both normoxic and hypoxic conditions. Moreover, PCAFs expressed higher level of PD-L1 under hypoxia, which was not observed in PFs. These results suggest that PCAFs may foster an immunosuppressive tumor microenvironment, particularly under hypoxic conditions.

Fibroblast Growth Factor Receptors (FGFRs) are a family of receptor tyrosine kinases that are widely expressed on cell surfaces and play essential roles in both embryonic development and adult tissue homeostasis. Accumulating evidence indicates that FGFR-driven oncogenesis is frequently associated with gene amplification, activating mutations, or chromosomal translocations across a wide range of tumor types. Aberrant FGFR signaling has been linked to numerous malignancies, including bladder, stomach, and lung cancers. Given their pivotal role, FGFRs represent attractive targets for anticancer therapies. Popiel et al. reported that CPL304100, a novel and potent inhibitor targeting FGFR1-3 kinase domains exhibited robust biological activity *in vitro*. CPL304100 could selective bind to the kinase domains of FGFR1/2/3, active FGFR signaling pathways and inhibit FGFR positive tumor cell growth*.* Additionally, therapeutic efficacy of CPL304100 was also observed in patient-derived xenograft (PDX) models. These findings highlighted CPL304100 as a potential new treatment for cancer.

Heterogeneity of CAFs have been demonstrated in multiple cancers. Here, Herch et al. provided a comprehensive overview of the heterogeneous populations and their functional roles of CAFs in head and neck squamous cell carcinoma (HNSCC). CAFs interact with tumor cells, immune cells and other components within the tumor microenvironment (TME) to promote HNSCC progression. Most studies demonstrate the predominant role of CAFs contributing to cancer development, while some studies highlight the presence of CAF subtypes that can restrain tumor growth. Although CAFs are potential target for treatment of cancers, there is no significant therapeutic advancement in HNSCC, underscoring the need for further mechanistic study to investigate the complex regulation of CAFs and develop effective therapeutic strategies.

Gastrointestinal (GI) cancers, including colorectal, gastric, liver, oesophageal, and pancreatic cancer, are a major cause of morbidity and mortality worldwide. Gastric cancer is one of the most aggressive malignancies, which is resistant to various cancer treatments. Accmulating studies identify CAFs as the leading factor contributing to an unfavorable tumor immune microenvironment and poor prognosis in gastric cancer patients. Ozmen et al. have summarized the current knowledge and perspective on the heterogeneity, protumorigenic function of CAFs and the underlying mechanisms in gastric cancer. Preventing the development and activation of CAFs by inhibiting their inducers and identifying markers that characterize these fibroblasts are potential therapeutic strategies for gastric cancer.

The interplay between the tumor microenvironment (TME) and tumor cells constitutes a sophisticated network that drives rapid cancer cell proliferation, metastasis, and therapeutic resistance. CAF plays a pivotal role in extracellular matrix (ECM) remodeling, promoting angiogenesis, secreting exosomes to communicate with other cells and modulating immune cell infiltration in the TME, thereby promoting tumor cell proliferation, metastasis and contributing to the resistance of chemotherapy. Dhungel et al. and Cao et al. provided the comprehensive reviews outlining both direct and indirect mechanisms of CAFs to promote tumor development and metastatic spread by direct physical interactions with tumor cells or secreting exosomes to modulate the biological phenotype of tumor cells. In addition, Piwocka et al.reviewed the detailed regulation of treatment response by CAFs. CAFs could modulate drug efflux, DNA repair and suppress apoptosis in tumor cells, leading to therapy resistance. These reviews shed light on targeting crosstalk between CAFs and other cells as potential therapies for cancer.

In conclusion, the contributions to this Research Topic highlight the critical significance of fibroblast in promoting cancer progression. The better understanding of the heterogeneity, protumoral function and the underlying mechanisms of CAFs will pave the way for the development of novel and effective cancer treatments.
